# Pathway-Based Analysis of Genome-Wide Association Data Identified SNPs in *HMMR* as Biomarker for Chemotherapy- Induced Neutropenia in Breast Cancer Patients

**DOI:** 10.3389/fphar.2018.00158

**Published:** 2018-03-13

**Authors:** Behzad Bidadi, Duan Liu, Krishna R. Kalari, Matthias Rubner, Alexander Hein, Matthias W. Beckmann, Brigitte Rack, Wolfgang Janni, Peter A. Fasching, Richard M. Weinshilboum, Liewei Wang

**Affiliations:** ^1^Division of Clinical Pharmacology, Department of Molecular Pharmacology and Experimental Therapeutics, Mayo Clinic, Rochester, MN, United States; ^2^Division of Biomedical Statistics and Informatics, Department of Health Sciences Research, Mayo Clinic, Rochester, MN, United States; ^3^Department of Gynecology and Obstetrics, University Hospital Erlangen, Friedrich-Alexander-University Erlangen-Nuremberg, Erlangen, Germany; ^4^Department of Gynecology and Obstetrics, Ludwig-Maximilians-Universität München, Munich, Germany; ^5^Department of Gynecology and Obstetrics, University Hospital Ulm, Ulm, Germany

**Keywords:** breast cancer, neutropenia, *HMMR*, *TNFSF13B*, GWAS

## Abstract

Neutropenia secondary to chemotherapy in breast cancer patients can be life-threatening and there are no biomarkers available to predict the risk of drug-induced neutropenia in those patients. We previously performed a genome-wide association study (GWAS) for neutropenia events in women with breast cancer who were treated with 5-fluorouracil, epirubicin and cyclophosphamide and recruited to the SUCCESS-A trial. A genome-wide significant single-nucleotide polymorphism (SNP) signal in the tumor necrosis factor superfamily member 13B (*TNFSF13B*) gene, encoding the cytokine B-cell activating factor (BAFF), was identified in that GWAS. Taking advantage of these existing GWAS data, in the present study we utilized a pathway-based analysis approach by leveraging knowledge of the pharmacokinetics and pharmacodynamics of drugs and breast cancer pathophysiology to identify additional SNPs/genes associated with the underlying etiology of chemotherapy-induced neutropenia. We identified three SNPs in the hyaluronan mediated motility receptor (*HMMR)* gene that were significantly associated with neutropenia (*p* < 1.0E-04). Those three SNPs were trans-expression quantitative trait loci for the expression of *TNFSF13B* (*p* < 1.0E-04). The minor allele of these *HMMR* SNPs was associated with a decreased *TNFSF13B* mRNA level. Additional functional studies performed with lymphoblastoid cell lines (LCLs) demonstrated that LCLs possessing the minor allele for the *HMMR* SNPs were more sensitive to drug treatment. Knock-down of *TNFSF13B* in LCLs and HL-60 promyelocytic cells and treatment of those cells with BAFF modulated the cell sensitivity to chemotherapy treatment. These results demonstrate that *HMMR* SNP-dependent cytotoxicity of these chemotherapeutic agents might be related to *TNFSF13B* expression level. In summary, utilizing a pathway-based approach for the analysis of GWAS data, we identified additional SNPs in the *HMMR* gene that were associated with neutropenia and also were correlated with *TNFSF13B* expression.

## Introduction

Breast cancer is the most common invasive cancer in women worldwide and approximately 1.3 million cases are diagnosed worldwide annually (Ferlay et al., 2015). Those patients with high-risk features require chemotherapy following surgical resection of the tumor. However, administration of chemotherapy regimens such as cytotoxic agents potentially leads to life-threatening adverse drug effects, of which the most common adverse event is chemotherapy-induced neutropenia (CIN) (Fontanella et al., 2014). This adverse event is common in breast cancer patients receiving adjuvant chemotherapy and affects more than 50% of patients (Holmes et al., 2002; Therasse et al., 2003). More than 20% of patients with breast cancer receiving adjuvant chemotherapy developed febrile neutropenia, which is a more severe, life-threatening adverse event (Do et al., 2015). CIN may lead to infection and hospitalization, which potentially results in compromised treatment because of the requirement for reduction in dose intensity of the chemotherapy regimen (Link et al., 2001; Fontanella et al., 2014; Agiro et al., 2016). To prevent CIN, co-administration of white blood cell growth factor such as the granulocyte colony-stimulating growth factor (G-CSF) is recommend with chemotherapy (Smith et al., 2015), and has shown benefit in patients with breast cancer who received chemotherapy (Holmes et al., 2002; Vogel et al., 2005; Agiro et al., 2016). However, considerable inter-individual variabilities in both risk and severity of CIN, and in the beneficial effect of G-CSF prophylaxis were found among breast cancer patients (Agiro et al., 2016). Sub-classification of patients at increased risk of CIN may allow for improved treatment modifications, such as G-CSF support, dose reduction, or use of chemotherapy regimens with a lower risk of myelosuppression. Clinical risk factors such as older age, poor nutrition and prior chemotherapy have been associated with increased risk of febrile neutropenia (Aapro et al., 2006). However, predictive models using these factors for risk of CIN have shown limited utility (Lyman et al., 2015). Other predictors for CIN risk are urgently needed which may help to improve the individualized care of breast cancer patients during adjuvant chemotherapy.

Germline genetic polymorphisms in patients with cancer have been associated with risk for chemotherapy-related toxicity, including CIN (Ulrich et al., 2003; Pinto et al., 2012). Using a candidate gene approach, previous studies have demonstrated that single-nucleotide polymorphisms (SNPs) in genes encoding drug-metabolizing enzymes and transporters were associated with the risk for CIN in breast cancer patients (Low et al., 2009; Yao et al., 2010; Okishiro et al., 2012; Tang et al., 2013). However, those studies included a limited number of SNPs/genes and relatively small sample sizes, which might explain the contradictory results among these studies — e.g., no SNPs/genes were replicated from one study to another even though the same chemotherapy regimens were administrated to those breast cancer patients. To our knowledge, no genome-wide association study (GWAS) for CIN has been reported. Compared to the candidate gene approach, GWAS is an “unbiased” approach that has the capability of revealing novel genetic variants associated with drug response (Weinshilboum and Wang, 2004; Wang et al., 2011; Motsinger-Reif et al., 2013). We have previously performed GWASs for adverse events in breast cancer patients treated with aromatase inhibitors and identified novel genetic variants associated with musculoskeletal pain (Ingle et al., 2010) and bone fractures (Liu et al., 2014). Recently, we performed a GWAS for CIN in 3,252 women with early stage breast cancer who received adjuvant chemotherapy. Novel SNP signals that were genome-wide significantly associated with CIN were identified in that GWAS. That GWAS will be reported in detail elsewhere.

Although GWAS has been very successful for identifying novel and statistically significant SNPs that were associated with phenotypes, many SNPs/genes that have biological significance could be overlooked in GWAS since those SNPs do not meet the threshold to reach genome-wide significance (Wang et al., 2007, 2010). To overcome this problem, pathway-based approaches, which consider prior knowledge of genes and pathways contributing to the phenotype of interest has been developed to provide additional insight into the interpretation of GWAS data on complex phenotypes (Wang et al., 2007; Ramanan et al., 2012). This approach has been successfully used to analyze and interpret GWAS data for many clinical phenotypes (Torkamani et al., 2008; Baranzini et al., 2009; Holmans et al., 2009; Ballard et al., 2010; Jia et al., 2010)

In the present study, we took advantage of the GWAS data we generated for CIN in breast cancer patients, and performed pathway-based analysis to identify SNPs/genes that might be associated with CIN. The SNPs identified by this pathway-based analysis were pursued by functional characterization to help us understand underlying mechanisms.

## Materials and Methods

### Patients and the Clinic Trial

Patients in this study were recruited to the SUCCESS-A trial (ClinicalTrials.gov Identifier: NCT02181101), a randomized Phase III study of response to the treatment of early primary breast cancer with adjuvant therapy after surgical resection (Rack et al., 2014; Widschwendter et al., 2015). All patients initially received three cycles of epirubicin, 5-fluorouracil (5-FU), and cyclophosphamide. Patients were then randomized to either receive three cycles of either gemcitabine in addition to docetaxel or docetaxel alone. A second randomization consisted of zoledronate at the conclusion of treatment for either 2 or 5 years. This trial recruited 3754 patients over 18 months in 251 medical centers in Germany, and accrual ended in March 2007. The main study and all pre-specified translational research projects, including the one reported here, were approved by all of the responsible ethics committees and were conducted in accordance with the Declaration of Helsinki. This study was also reviewed and approved by the Mayo Clinic Institutional Review Board. All patients gave written informed consent.

### Case Definition for Neutropenia and GWAS

Cases were defined as patients who had at least one of the following events: leukopenia, neutropenia or febrile neutropenia, during cycles one to three of chemotherapy with epirubicin, 5-fluorouracil (5-FU), and cyclophosphamide. Cases were required to meet the toxicity level of Grade 3 or 4 based on the National Cancer Institute (NCI)’s Common Terminology Criteria for Adverse Events v3.0. A total of 1648 patients fulfilled the criteria for cases. Other patients served as controls for the GWAS analysis. Patient DNA samples were genotyped on the Illumina HumanOmniExpress-12v1 G FFPE array (Illumina, San Diego, CA, United States). After remove of 5 related patients and 9 patients who were non-Caucasian (Asian), a total of 3,252 patients (1,635 controls vs. 1,617 cases) who passed QC for genotyping and whose covariate information (Supplementary Table S1) was available were included in the GWAS analysis. Imputation was performed based on 1000 Genomes Project data (Howie et al., 2012). Genotyped SNPs which had a *p-*value < 1.0E-05 in the initial GWAS were used as input SNPs to perform imputation. Genotyping and imputation are described in detail in the Supplementary Methods. Data that were generated in the SUCCESS-A trial, including genotype and clinical phenotype data, as well as a further descriptions of materials can be found in the NCBI database of Genotypes and Phenotypes (dbGaP Study Accession: phs000547.v1.p).

### Selection of Pathways, Genes and SNPs

Genes involved in pathways of (1) the pharmacokinetics (PK) and pharmacodynamics (PD) for the chemotherapeutic drugs used in the SUCCESS-A trial, cyclophosphamide, epirubicin and 5-FU were included in this analysis. These candidate genes were identified using the PharmGKB database^1^. Genes involved in the PK/PD of doxorubicin, an analog of epirubicin, were selected because epirubicin is not included in the PharmGKB. (2) Pathways that were shown to be aberrant in breast cancer according to The Cancer Genome Atlas (TCGA) data were included in the analysis (The Cancer Genome Atlas Network, 2012). Those pathways were the PI3K, mTOR, p53, apoptosis, and checkpoint signaling pathways. Genes in these pathways were identified using the Kyoto Encyclopedia of Genes and Genomes (KEGG) database (Kanehisa and Goto, 2000). (3) The PAM50 genes which have been shown to be effective in predicting disease prognosis (Nielsen et al., 2010) were also included. (4) Finally, genes studied in our laboratory shown to be functionally relevant in the AKT/mTOR signaling pathways in chemotherapy response (Pei et al., 2009; Hou et al., 2014; Yu et al., 2017) and toxicity (Ingle et al., 2010) were also added to the analysis.

SNPs within 50 kb up- and down-stream, and across the selected genes as described above were included in this analysis. SNPs that were located > 50 kb away from a gene might also affect gene transcription and function. Those SNPs will be overlooked in this study. Genotyping information for SNPs in those pathway genes in the SUCCESS-A patients was obtained from our GWAS genotyping data. Imputation was performed based on 1000 Genomes Project data (Howie et al., 2012). See Supplementary Methods for details.

### Association of SNPs With Neutropenia

The analysis for association of SNPs with neutropenia was based on conditional logistic regression to account for the matched design. SNP genotypes were coded as additive effects on the log OR by coding 0, 1, or 2 for the minor allele count. A likelihood ratio test with one degree of freedom for each SNP was then determined. The primary covariates (Supplementary Table S1) used to match cases and controls were controlled in the conditional logistic regression. Association analyses were performed in R^2^, SAS (SAS Institute Inc.) and PLINK. Logistic regression models were used to calculate Odds ratios (ORs), 95% confidence intervals (CIs), and *p*-values, following corrections for multiple testing.

### eQTL Analysis

Single-nucleotide polymorphisms of interest that were associated with neutropenia were further subjected to expression quantitative trait loci (eQTL) analysis using our lymphoblastoid cell line (LCL) data sets, which have been described in detail in our previous studies (Li et al., 2008, 2014; Niu et al., 2010; Matimba et al., 2014; Liu et al., 2017). The Human Variation Panel of LCLs from 96 European-American (EA), 95 African-American (AA), and 96 Han Chinese-American (HCA) healthy subjects were obtained from the Coriell Institute. Those LCLs are B-lymphocytes that were immortalized by Epstein Barr Virus (EBV) infection. Genome-wide genotype data and mRNA expression data for these LCLs have been generated in our laboratory by using the Illumina 550K and 510S SNP BeadChips (Illumina, San Diego, CA, United States), and the Affymetrix Human Genome U133 Plus 2.0 GeneChip arrays (Affymetrix, Santa Clara, CA, United States), respectively. Genotype and gene expression data were deposited in the National Center for Biotechnology Information Gene Expression Omnibus (GEO accession: GSE23120). Expression array data were normalized on a log2 scale using GCRMA (Wu et al., 2004). The normalized expression data were then regressed on gender. Partial Pearson correlations were used to quantify the association between SNPs and mRNA expression. Since the LCLs were from multiple races/ethnic groups, before completing the genetic association analysis, population stratification was assessed using the method developed by Price et al. (2006) as described in detail previously (Niu et al., 2010). These partial correlations were tested using a Wald test. False discovery *q*-values (Storey and Tibshirani, 2003) were also computed for each test.

### Cell Culture and Cytotoxicity Assays

Lymphoblastoid cell lines were cultured in RPMI 1640 media containing 15% fetal bovine serum (FBS). The human promyelocytic leukemia cell line, HL-60, was obtained from the American Type Culture Collection (ATCC, Manassas, VA, United States), and was cultured in RPMI 1640 media containing 10% FBS. Epirubicin (EPI) was purchased from Santa Cruz Biotechnology, Inc. (Santa Cruz, CA, United States). Mafosfamide (MFF) was purchased from Toronto Research Chemicals Inc. (Toronto, Canada). MFF can spontaneously decompose to 4-hydroxy-cyclophosphamide, the active metabolite of cyclophosphamide, when added in culture media (Mazur et al., 2012). Fluorouracil (5-FU) was purchased from Sigma-Aldrich (St. Louis, MO, United States). Cytotoxicity assays with LCLs and HL-60 cells were performed with the CellTiter 96^®^ Aqueous Non-Radioactive Cell Proliferation Assay (Promega Corporation, Madison, WI, United States) in 96 well plates (Corning, Corning, NY, United States) at a density of 5 × 10^5^ cells/mL (100 μL/well). 10 μL of 5-FU (500–0.01 μM), epirubicin (10–0.0005 μM), or mafosfamide (100–0.005 μM) were added into the wells and incubated at 37°C for 72 h. Plates were then added with 20 μL of MTS buffer and read in an Infinite M1000 PRO plate reader (Tecan AG, Switzerland) after incubation for 3 h. Relative cell viability was then plotted against drug concentration to derive a cytotoxicity curve. Experiments were independently repeated at least twice with triplicate wells for each treatment. Independent *t*-test was used to determine statistical significance.

### mRNA Quantification

Messenger RNA levels were quantified by quantitative reverse transcription PCR (qRT-PCR) using Power SYBR^TM^ Green RNA-to-CT^TM^ 1-Step Kit (Applied Biosystems Inc., Foster City, CA, United States). Total RNA was extracted from cells by the RNeasy Plus Mini Kit (QIAGEN, Germany). A total of 100 ng of total RNA was used for each reaction. Specific primers for *TNFSF13B* mRNA were purchased from Integrated DNA Technologies (Coralville, IA, United States). The qRT-PCR reaction was performed using the Stratagene Mx3005P Quantitative Real-Time PCR detection system (Agilent Technologies, Santa Clara, CA, United States). Glyceraldehyde 3-phosphate dehydrogenase (GAPDH) was used as an internal control.

### *TNFSF13B* KD and Ligand Treatment for Cytotoxicity Assays

LCLs and HL-60 cells were incubated at a density of 1 × 10^6^ cells/mL in RPMI 1640 with 5% charcoal-stripped FBS for 24 h followed by serum free medium for an additional 24 h. For *TNFSF13B* KD, cells were transfected with negative control and *TNFSF13B* siRNAs (Dharmacon, Lafayette, CO, United States) separately, and incubated in RPMI 1640 with 5% charcoal-stripped FBS overnight before cytotoxicity assay. For TNFSF13B ligand (BAFF) treatment, recombinant human BAFF (Cell Signaling Technology, Danvers, MA, United States) reconstituted in PBS containing 200 μM dithiothreitol (DTT) and 0.02% bovine serum albumin was added to the cell culture media at a final concentration of 1 μg/mL and were incubated for 24 h. Vehicle treatment that included DTT and BSA but without BAFF was performed as control. After KD and BAFF treatment, cytotoxicity assays were performed as describe above. Experiments were repeated independently three times.

## Results

A summary of the patients’ characteristics is listed in the Supplementary Table S1. A GWAS for CIN in women with early stage breast cancer who were enrolled in the SUCCESS-A trial has been completed. The Manhattan plot of the GWAS for CIN in women with breast cancer during three cycles of epirubicin, 5-FU, and cyclophosphamide treatment, is shown in Supplementary Figure S1. Two genome-wide significant (*p* < 5.0E-08) SNP signals were identified, including one that mapped to the *TNFSF13B* gene on chromosome 13, with lowest *p-*value of 2.4E-08. *TNFSF13B* encodes a cytokine known as B-cell activating factor (BAFF) which plays an important role in the proliferation and differentiation of B-cells (Mackay and Browning, 2002).

Taking advantages of the existing GWAS data, we utilized a pathway-based approach by considering prior knowledge of these drugs and the disease, to identify additional SNPs/genes that might be associated with neutropenia.

### Pathway-Based Genes and SNPs

Ninety-five candidate genes involved in the PK and PD pathways of the chemotherapeutic drugs used in the SUCCESS-A trial were identified (Supplementary Table S2). In addition, 311 genes were identified in pathways that were shown to be aberrant in breast cancer according to TCGA data (The Cancer Genome Atlas Network, 2012) were included (Supplementary Table S3). The 50 genes in the PAM50 gene list (Supplementary Table S4) which have been shown to be effective in predicting disease prognosis (Nielsen et al., 2010), and 12 genes that had been identified in previous cancer studies in our laboratory (Supplementary Table S5) were also added to the analysis. A summary of the pathways, number of genes and SNPs is listed in **Table 1**. Several genes were included in multiple pathways and overlapping SNPs were removed during the follow up analysis. Finally, a total of 325, 934 SNPs were included in the analysis for association with CIN in breast cancer patients.

**Table 1:** Pathways and numbers of genes and single-nucleotide polymorphisms (SNPs) involved in pathways included in the analysis and the number of SNPs that were associated with neutropenia.

Pathway	Pathway gene source	Number of pathway genes	Number of SNPs in pathway genes	Number of SNPs associated with neutropenia (*p* < 1.0E-04)
Cyclophosphamide PK/PD	PharmGKB	40	17,548	1
Doxorubicin PK/PD	PharmGKB	30	18,879	5
5-Fluorouracil PK/PD	PharmGKB	28	21,063	1
Apoptosis	TCGA/KEGG	72	47,321	1
Checkpoint	TCGA/KEGG	46	27,072	4
P53 signaling	TCGA/KEGG	88	53,318	24
mTOR signaling	TCGA/KEGG	98	69,486	17
PI3K signaling	TCGA/KEGG	100	63,853	15
PAM50	PAM50	50	31,550	0
AKT/mTOR signaling	“In house”	12	7,394	0


**PK, pharmacokinetics; PD, pharmacodynamics; PAM50, prediction analysis of microarray 50; PharmGKB, www.pharmgkb.org; TCGA, The Cancer Genome Atlas; KEGG, Kyoto encyclopedia of genes and genomes; “In house” means the pathway and gene were previously characterized in this laboratory.**

### SNP Associations With Neutropenia and eQTL Analysis

**FIGURE 1:**
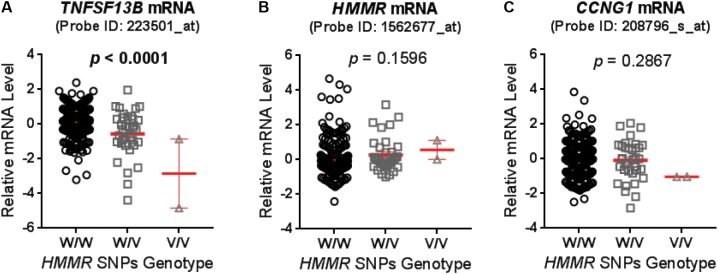
Expression quantitative trait loci (eQTL) analysis for hyaluronan mediated motility receptor (*HMMR*) single-nucleotide polymorphisms (SNPs) (rs299293, rs299313, and rs299314) in lymphoblastoid cell lines (LCLs). These three *HMMR* SNPs belong to a same haplotype and their genotypes were same in each LCLs. The mRNA level of **(A)** *TNFSF13B*, **(B)** *HMMR*, and **(C)** *CCNG1* were grouped based on the genotype of *HMMR* SNPs, and compared by one-way ANOVA. A *p*-value < 0.05 was considered statistically significant. The mRNA level was measured by the Affymetrix U133 Plus 2.0GeneChip expression array with specific probes as specified. Values are mean ± SEM for mRNA level in LCLs with wild type (W/W, *n* = 247), heterozygous variant (W/V, *n* = 35) and homozygous variant (V/V, *n* = 2) genotypes (showed in red line and error bars), respectively.

Genotypes of selected SNPs in the pathway genes were associated with the CIN phenotype in patients recruited in the SUCCESS-A trial. A total of 35 SNPs were identified that were associated with CIN with a *p-*value < 1.0E-04 (**Table 2**). The associations listed in **Table 2** are “nominal” and have not been corrected for multiple comparisons because the purpose was to identify SNPs/genes for functional validation. None of those SNPs was located in an exon or was a splicing variant. We then set out to determine whether those SNPs were either *cis*- or *trans*-eQTLs. To do that, we performed eQTL analysis using the 300 LCLs for which genome-wide SNP genotype and mRNA expression data had been generated in our lab (Niu et al., 2010). Six SNPs with very low MAFs (<0.010) were not applicable for eQTL analysis in those 300 LCLs because the number of LCLs with the variant SNP allele was too small to compare with LCLs with WT genotypes (**Table 2**). For other SNPs that underwent eQTL analysis, none SNPs were *cis*-eQTLs. However, we identified nine SNPs that were *trans*-eQTLs (**Table 2**). Interestingly, three SNPs, rs299293, rs299313, and rs299314, which were located in the hyaluronan mediated motility receptor (*HMMR)* gene, were *trans*-eQTLs for *TNFSF13B* (**Figure 1A** and Supplementary Table S6), one of the “top” hits identified in the original GWAS for neutropenia (Supplementary Figure S1). These 3 *HMMR* SNPs were in tight linkage disequilibrium (LD) (*r*^2^ > 0.87) and belonged to the same haplotype. The genotypes for those 3 SNPs in an LCL were matched – e.g., an LCL with homozygous variant genotype for one of those 3 SNPs was also homozygous variant genotype for the other two SNPs. These 3 *HMMR* SNPs were imputed SNPs in the pathway-based analysis, and they were excluded from the GWAS because none of the genotyped SNPs in LD with these three SNPs had a *p-*value < 1.0E-05, the cut-off value for input SNPs to perform imputation for GWAS analysis. The rs299293 SNP was located in intron 12, and the rs299313 and rs299314 SNPs were in intron 16 of *HMMR*. Those 3 SNPs mapped to the 3′ end of the cyclin G1 (*CCNG1*) gene which is involved in the p53 signaling pathway. However, those three SNPs were not eQTLs for either *HMMR* (**Figure 1B**) or *CCNG1* (**Figure 1C**) mRNA expression in LCLs.

**Table 2:** Single-nucleotide polymorphisms (SNPs) associated with risk for chemotherapy-induced neutropenia (*p* < 1.0E-04) in women with breast cancer recruited to the SUCCESS-A trail (*n* = 3252) and expression quantitative trait loci (eQTL) analysis in lymphoblastoid cell lines (LCLs).

SNP rs ID	Chr.	Common allele	Minor allele	OR	MAF	*p*-value	Gene	Pathway	eQTL in LCLs
rs144991623	13	C	T	69.8	0.002	5.98E-06	*FOXO1*	Checkpoint, PI3K	NA
rs9389568	6	T	C	0.59	0.049	7.10E-06	*PERP*	P53	Yes
rs8110364	19	G	A	3.09	0.011	2.23E-05	*PIK3R2*	mTOR, PI3K, P53	Yes
rs56022120	19	C	T	3.12	0.011	2.29E-05	*PIK3R2*	mTOR, PI3K, P53	No
rs150688309	19	C	G	2.87	0.013	2.67E-05	*PIK3R2*	mTOR, PI3K, P53	No
rs58695150	19	C	T	3.07	0.011	3.15E-05	*PIK3R2*	mTOR, PI3K, P53	Yes
rs299293	**5**	**C**	**T**	**1.36**	**0.133**	**3.19E-05**	***HMMR/CCNG1***	**P53**	**Yes**
rs138602176	19	G	A	2.97	0.011	3.24E-05	*PIK3R2*	mTOR, PI3K, P53	No
rs148235907	19	G	A	3.06	0.011	3.35E-05	*PIK3R2*	mTOR, PI3K, P53	No
rs117951771	19	C	T	3.06	0.011	3.37E-05	*PIK3R2*	mTOR, PI3K, P53	No
**rs299314**	**5**	**T**	**C**	**1.35**	**0.138**	**3.60E-05**	***HMMR/CCNG1***	**P53**	**Yes**
**rs299313**	**5**	**G**	**A**	**1.35**	**0.139**	**3.72E-05**	***HMMR/CCNG1***	**P53**	**Yes**
rs115457081	2	G	A	5.04	0.012	3.89E-05	*IRS1*	mTOR	No
rs117341846	19	C	T	3.03	0.011	3.93E-05	*PIK3R2*	mTOR, PI3K, P53	No
rs4968187	17	C	T	0.10	0.004	4.09E-05	*TP53*	5FU, Apoptosis, Checkpoint, Doxorubicin, PI3K, P53	NA
rs142244113	19	C	T	1.72	0.039	4.59E-05	*INSR*	mTOR	No
rs79430272	19	C	T	2.94	0.011	4.80E-05	*PIK3R2*	mTOR, PI3K, P53	No
rs118129530	19	G	A	3.00	0.011	4.82E-05	*PIK3R2*	mTOR, PI3K, P53	No
rs77769901	14	A	G	0.31	0.012	4.99E-05	*CCNK*	P53	No
rs145623321	19	C	T	3.01	0.010	5.35E-05	*PIK3R2*	mTOR, PI3K, P53	No
rs148013902	19	C	T	3.01	0.010	5.46E-05	*PIK3R2*	mTOR, PI3K, P53	No
rs55633228	19	C	T	3.00	0.010	5.59E-05	*PIK3R2*	mTOR, PI3K, P53	No
rs141084494	22	G	A	0.05	0.003	6.26E-05	*RBX1*	Checkpoint	NA
rs41412545	19	C	A	1.70	0.038	6.36E-05	*INSR*	mTOR	No
rs3805945	6	T	C	1.68	0.040	6.81E-05	*PPP2R5D*	mTOR	No
rs117101815	6	G	T	0.63	0.051	7.17E-05	*PERP*	P53	Yes
rs9402944	6	G	T	0.63	0.051	7.20E-05	*PERP*	P53	Yes
rs181501757	17	G	A	4.77	0.008	7.75E-05	*TOP2A*	Doxorubicin	NA
rs149212925	12	A	G	0.00	0.001	8.33E-05	*NOS1*	Doxorubicin	NA
rs112783657	21	C	T	0.49	0.027	8.65E-05	*CBR3*	Doxorubicin	No
rs74743371	21	G	T	0.49	0.027	8.68E-05	*CBR3*	Doxorubicin	No
rs117458836	10	G	A	2.53	0.014	8.85E-05	*CYP2C8*	Cyclophosphamide	No
rs118088833	11	C	T	0.26	0.009	8.99E-05	*TP53AIP1*	P53	NA
rs112242273	3	C	T	0.52	0.038	9.58E-05	*GNL3*	Checkpoint, P53	No
rs78428806	6	G	A	0.63	0.048	9.79E-05	*PERP*	P53	Yes


**Chr., chromosome; OR, odds ratio; MAF, minor allele frequency; NA, not applicable, because SNP MAF is too low to find LCLs with variant SNP allele*. The three SNPs in HMMR gene were highlighted in bold.*

### *HMMR* SNP-Dependent Cytotoxicity

**FIGURE 2:**
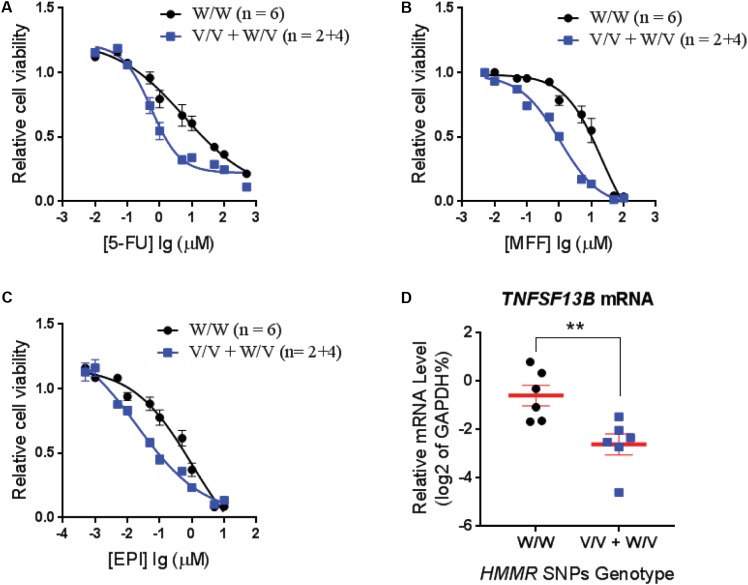
Cytotoxicity for LCLs with different genotypes for *HMMR* SNPs. Average cytotoxicity for **(A)** fluorouracil (5-FU), **(B)** mafosfamide (MFF), and **(C)** epirubicin (EPI) in LCLs with W/W genotypes (*n* = 6) vs. LCLs with V/V and W/V genotypes (*n* = 2+4). Cell viability was tested by MTS assay after 72 h of drug exposure. Relative cell viability was plotted against drug concentration to derive a cytotoxicity curve. Cytotoxicity for each LCL was averaged from triplicate determinations. Values represent mean ± SEM of LCLs in each genotype group. **(D)** *TNFSF13B* mRNA level in LCLs that are used for cytotoxicity assay were determined by qRT-PCR and grouped based on the *HMMR* SNP genotype. Values are mean ± SEM and compared by unpaired *t-*test. A *p*-value < 0.05 was considered statistically significant. ^∗∗^*p* < 0.01.

To confirm that the SNPs in the *HMMR* gene were associated with neutropenia, we performed cytotoxicity assays for drugs used in the SUCCESS-A trial using the LCLs with WT and variant genotypes for the *HMMR* SNPs. Six LCLs with variant SNP genotypes and six with WT genotype were chosen randomly for cytotoxicity assay. Since only two LCLs with homozygous variant genotypes for those SNPs were available, four LCLs heterozygous variant for those SNPs were included in the variant group. To confirm genotypes for those three SNPs in *HMMR* which were imputed based on the 1K Genome data, DNA sequencing was performed for those SNPs in these 12 LCLs that were later used in cytotoxicity assays. We found that LCLs possessing the variant *HMMR* SNP genotypes were more sensitive to all three agents, 5-FU, MFF, and EPI (**Figures 2A–C**). This result was consistent with our clinical observations in patients treated with these drugs. Patients with the variant SNP genotype had a higher risk of neutropenia compared to patients with the WT SNP genotype (OR = 1.36) (**Table 2**). The eQTL analysis for those *HMMR* SNPs indicated that the SNPs were *trans*-eQTLs for *TNFSF13B* and variant SNP genotypes were associated with decreased *TNFSF13B* mRNA level (**Figure 1A**). To further determine *TNFSF13B* mRNA levels in those 12 LCLs that were used for cytotoxicity assays, qRT-PCR for *TNFSF13B* mRNA were performed with total RNA extracted from those LCLs. The average mRNA level of *TNFSF13B* in six LCLs with variant SNP alleles was significantly decreased compared with six LCLs with WT genotype (*p* = 0.006) (**Figure 2D**), a result consistent with the eQTL analysis (**Figure 1A**). The mRNA levels of *TNFSF13B*, *HMMR*, and *CCNG1* in 12 LCLs that were determined by mRNA microarray again indicated a decrease in the *TNFSF13B* mRNA in LCLs with variant SNP genotypes but no significant changes in the *HMMR* and *CCNG1* mRNA levels (Supplementary Figure S2).

### TNFSF13B and Chemo-Drug Sensitivity

**FIGURE 3:**
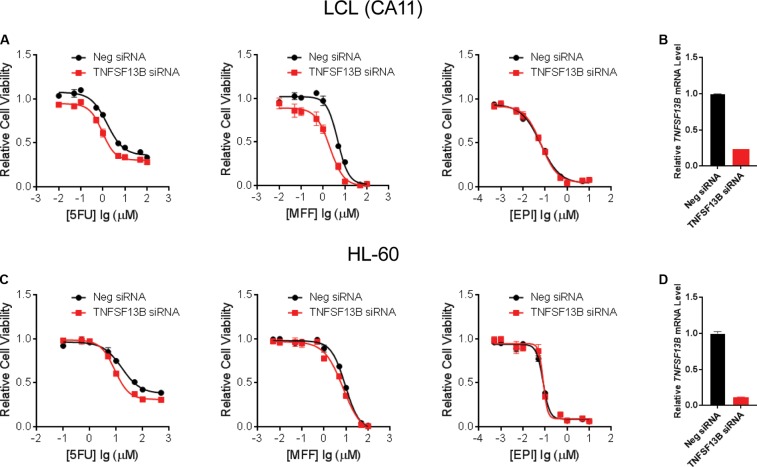
Cytotoxicity for LCLs and HL-60 after *TNFSF13B* Knockdown. **(A)** Cytotoxicity for one of the LCLs, CA11, treated with 5-FU (left), MFF (middle), and EPI (right) after knockdown (KD) of *TNFSF13B*. **(B)** *TNFSF13B* mRNA level in LCL CA11 after 24 h of transfection of *TNFSF13B* siRNA vs. non-targeting scrambled control (Neg.) siRNA. **(C)** Cytotoxicity for HL-60 cells treated with 5-FU (left), MFF (middle) and EPI (right) after knockdown (KD) of *TNFSF13B*. **(D)** *TNFSF13B* mRNA level in HL-60 after 24 h of transfection of *TNFSF13B* siRNA vs. non-targeting scrambled control (Neg.) siRNA. Values represent mean ± SEM from triplicate determinations.

**FIGURE 4:**
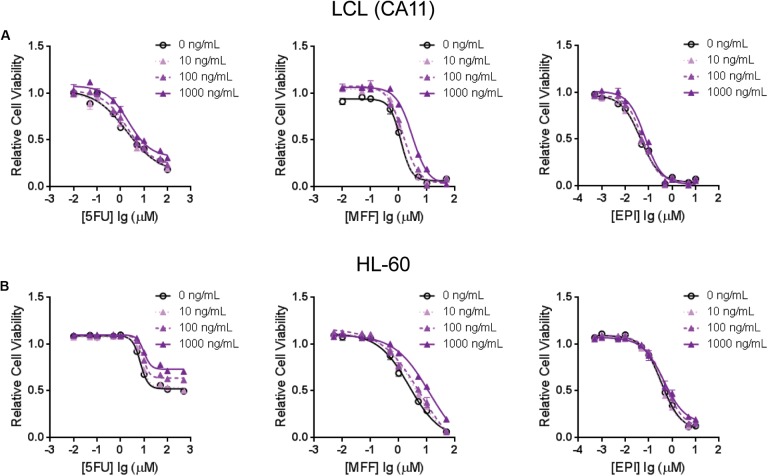
Cytotoxicity assays for **(A)** one of the LCLs, CA11, and for **(B)** HL-60 that were treated with 5-FU (left), MFF (middle) and EPI (right) with incubation of human recombinant B-cell activating factor (BAFF) at different doses. Values represent mean ± SEM from triplicate determinations.

To further demonstrate that *TNFSF13B* expression was important for the sensitivity to these chemotherapeutic agents, we performed a cytotoxicity assay in an LCL with relatively high *TNFSF13B* expression after knock-down (KD) of *TNFSF13B*. The same cytotoxicity assay was also performed with a human promyelocytic leukemia cell line, HL-60, a cell representative of promyelocytes which can differentiate to myelocytes, the major components of white blood cells. These cells were more sensitive to 5-FU and MFF treatment, but not EPI treatment, in both cell lines (**Figures 3A,C**) after *TNFSF13B* KD (**Figures 3B,D**) when compared with the negative control KD. Since *TNFSF13B* encodes a cytokine known as BAFF, we also performed cytotoxicity assays in the LCL and HL-60 cells with BAFF treatment. Both the LCL and HL-60 that were treated with recombinant human BAFF showed dose-dependent resistance to 5-FU and MFF treatment (**Figures 4A,B**, left and middle) but were not significantly changed by EPI treatment (**Figures 4A,B**, right).

We did not observe significant changes in EPI sensitivity in both LCL and HL-60 cells after *TNFSF13B* KD or BAFF treatment, while LCLs with variant SNP genotypes were more sensitive to EPI (**Figure 2C**). This might indicate additional mechanisms beyond *TNFSF13B* expression that are related to those SNP genotypes could contribute to EPI cytotoxicity. Although the *HMMR* SNPs were *trans*-eQTLs for *TNFSF13B*, we did not observe any correlation between *HMMR* and *TNFSF13B* mRNA expression based on the mRNA data from the 300 LCLs. We also measured *TNFSF13B* mRNA level after KD of *HMMR* and *CCNG1*, but no change in *TNFSF13B* mRNA was observed (data not shown). The HMMR mRNA level was also quantified after LCLs were treated with 5-FU, EPI, and MFF at the concentration of their IC_50_s, respectively. Compared to vehicle treatment, HMMR mRNA level (relative to GAPDH) was not significantly changed after LCLs were exposed to 5-FU (50 μM) and/or EPI (0.1 μM). Although HMMR mRNA levels were slightly decreased (∼15%) in LCLs after exposure to MFF, no significant differences were observed between WT and variant LCLs (data not shown). The molecular mechanism for SNPs in *HMMR* that was associated with *TNFSF13B* expression needs to be further explored.

In summary, this series of functional genomic experiments confirmed that three SNPs in the *HMMR* gene were associated with the cytotoxicity of drugs used in the SUCCESS-A trial, an effect which is probably mediated through the expression of *TNFSF13B*.

## Discussion

In the present study, we took advantage of GWAS data that we had generated in the SUCCESS-A trial, and utilized a pathway-based approach to identify additional biomarkers and underlying biology for CIN in breast cancer patients. Specifically, SNPs in genes involved in PK and PD pathways for the chemotherapy agents that were used to treat patients, and were involved in disease risk were associated with neutropenia events. We identified additional SNPs with low *p*-value that were associated with CIN, including SNPs in *HMMR* that are *trans*-eQTLs for *TNFSF13B*, one of the genome-wide significant signals identified in the original GWAS (Supplementary Figure S1). The variant *HMMR* SNP genotype was associated with decreased *TNFSF13B* expression (**Figure 1A**) and higher risk of CIN (**Table 2**). To further eliminate potential false positives results and to understand the underlying biology of these SNPs, we performed functional studies to further validate the association results. We demonstrated that LCLs carrying variant genotypes for the *HMMR* SNPs displayed decreased expression of *TNFSF13B*, and were more sensitive to treatment with 5-FU, mafosfamide and epirubicin (**Figure 2**). Further cytotoxicity assays conducted with *TNFSF13B* KD and TNFSF13B cytokine (BAFF) treatment in both LCL and HL-60 promyelocytic cells suggested that the modulation of *TNFSF13B* expression altered sensitivity to those chemotherapy agents (**Figures 3**, **4**). This study not only enabled us to discover additional SNPs associated with CIN, but also indicated an association with *TNFSF13B* expression.

The three *HMMR* SNPs that were identified by our pathway-based analysis were missed in the GWAS for CIN, probably because those SNPs did not directly contribute to CIN in breast cancer patients but rather through the expression of TNFSF13B. The *HMMR* gene encodes HMMR which is involved in cell motility. *HMMR* has been implicated in the pathogenesis of tumors like breast cancer and inflammatory disorders like osteoarthritis (Misra et al., 2015). We have demonstrated that SNPs within *HMMR* are associated with risk for CIN and that those SNPs are also *trans*-eQTLs for TNFSF13B expression. However, KD of HMMR did not directly affect TNFSF13B expression in the cell lines that we tested. *HMMR* has not been shown to be involved in the mechanism of treatment related neutropenia and the mechanism for how it might be associated with TNFSF13B expression is unclear and should be further explored. The association between *HMMR* SNPs and CIN in breast cancer patients needs to be further validated in an independent data set.

*TNFSF13B* encodes a cytokine known as BAFF, which is synthesized primarily by myeloid cells and is required for survival of B-cells. It is up-regulated in autoimmune diseases and various B-cell malignancies (Shivakumar and Ansell, 2006). Although it is involved primarily in B-cell survival, there has been increasing evidence that it may also be involved in reverse signaling in the activation of myeloid cells as well (Chang et al., 2006; Jeon et al., 2010). The role of this growth factor in the activation of myeloid cells is not fully understood and there is conflicting evidence that it is not involved in myeloid cell activation (Nys et al., 2013). Previous studies reported that serum BAFF levels were increased in patients who developed late onset neutropenia after therapy with rituximab (Terrier et al., 2007). However, genetic variation associated with baseline *TNFSF13B* level has not been reported to predispose patients to CIN. Our pathway-based analysis identified variant SNP genotypes in the *HMMR* gene that were associated with decreased *TNFSF13B* expression at baseline and with higher risk of CIN. The potential role of *TNFSF13B* is not only activating B-cells but also myeloid cells, a potential mechanism for how changes in expression of this gene might predispose patients to treatment related neutropenia. The mechanism of *TNFSF13B* expression in association with neutropenia is unclear and needs to be elucidated.

In addition to the *HMMR* SNPs, the pathway-based analysis also identified other SNPs that were significantly associated with CIN in breast cancer patients. However, those SNPs had a very low MAF (≤0.051) (**Table 2**), thus we were unable to perform functional validation for those SNPs using our 300 LCLs. Those SNPs were included in our pathway-based analysis because they are located in genes which are involved in the mTOR, PI3K, P53, and checkpoint pathways (**Table 2**), pathways have been found to be aberrant in breast cancer (The Cancer Genome Atlas Network, 2012). A recent study reported that common genetic variants for breast cancer risk were negatively associated with CIN (Dorling et al., 2016). Although that study used different criteria from ours for selection of SNPs/genes that correlated with breast cancer risk, they also found that the P53 signaling pathway was the most enriched pathway associated with CIN (Dorling et al., 2016). Using a candidate SNPs/genes approach, SNPs in the *TP53* and *MDM2* genes, two most important genes in the P53 signaling pathway, were found to be associated with CIN in Japanese breast cancer patients (*n* = 216) (Okishiro et al., 2012). Of note, the *HMMR* SNPs were included in our pathway-based analysis because those SNPs are located in a gene that is involved in the P53 signaling pathway (**Table 2**). It might be interesting to investigate the relationship between the P53 pathway, TNFSF13B and CIN in future studies.

Several studies have focused on the SNPs/genes involved in the cyclophosphamide PK pathway for associations with CIN in breast cancer patients. These studies reported that SNPs in *GSTP1* (Yao et al., 2010), *ABCC4* (Low et al., 2009) and *CYP3A5* (Tang et al., 2013) were associated with CIN in breast cancer patients. These studies did not include SNPs in *CYP2C8* while our analysis identified a SNP in *CYP2C8* that was associated with CIN (**Table 2**). Together, those studies indicate that genetic polymorphisms in the cyclophosphamide PK pathway may be associated with CIN in breast cancer patients. In addition to anti-cancer drugs, neutropenia can be also induced by other drugs such as clozapine, a dibenzodiazepine antipsychotic agent used for the pharmacotherapy of schizophrenia (Alvir et al., 1993; Atkin et al., 1996). Pharmacogenomic studies on clozapine-induced agranulocytosis or neutropenia have often identified SNPs/genes in the clozapine PK pathway and immune response genes (Athanasiou et al., 2011; Goldstein et al., 2014; Saito et al., 2016; Legge et al., 2017; van der Weide et al., 2017), a similar scenario to pharmacogenomic studies on CIN in breast cancer patients.

In summary, using a pathway-based approach for analyzing GWAS data, we identified additional genetic makers for CIN in breast cancer patients, including SNPs in the HMMR gene. Further functional genomic studies indicated that *HMMR* SNP-dependent sensitivity to chemotherapy is probably dependent on the expression of *TNFSF13B*.

## Author Contributions

BB and DL conducted the experiments and analyzed the data, and drafted the manuscript. KK did the pathway analysis, genes/SNPs selection, and association study with clinical phenotype. MR, AH, MB, BR, WJ, and PF recruited patients and measured the clinical phenotypes. RW and LW were responsible for the concept generation and entire study design.

## Conflict of Interest Statement

BB is a Merck & Co., Inc. employee. The other authors declare that the research was conducted in the absence of any commercial or financial relationships that could be construed as a potential conflict of interest.

## Footnotes

1 www.pharmgkb.org

2 http://www.r-project.org
