# Supplementary material for: Pathway-Based Analysis of Genome-Wide Association Data Identified SNPs in HMMR as Biomarker for Chemotherapy- Induced Neutropenia in Breast Cancer Patients
Source: Front Pharmacol. 2018 Mar 13;9:158. doi: 10.3389/fphar.2018.00158 (PMC5859084; doi:10.3389/fphar.2018.00158)
Supplement: Supplementary file 1 [file Presentation_1.pdf]

SUPPLEMENTARY MATERIALS

Pathway-Based Analysis of Genome-wide Association Data Identified SNPs in *HMMR* as Biomarker for Chemotherapy-induced Neutropenia in Breast Cancer Patients

Behzad Bidadi; Duan Liu; Krishna R. Kalari; Matthias Rubner; Alexander Hein; Matthias W. Beckmann; Brigitte Rack; Wolfgang Janni;Peter A. Fasching; Richard M. Weinshilboum; and Liewei Wang*

*** Correspondence:** Liewei Wang (wang.liewei@mayo.edu)

1 Supplementary Methods

Genotyping and Quality Control

Initially, blood collected at baseline before the first randomization was used to isolate DNA for GWAS genotyping assays. However, the quality control step identified quality issues (DNA degradation) with about one half of the DNA samples. A second blood drawn was performed for available patients. For those patients for whom a second blood drawn was not possible and whose DNA has quality issue, an Illumina FFPE restoration procedure was used for samples with quality problems. Of the samples, 1771 were from original blood, 1092 from redrawn blood samples and 459 from restored blood samples. DNA from 3304 patients was genotyped by the Center for Inherited Disease Research (CIDR) using the Illumina HumanOmniExpress-12v1 G FFPE array (Illumina, San Diego, CA), designed to human genome build 37. 3,252 genotyped subjects with covariate information were included in the analysis.

Imputation and Fine Mapping

1094 subjects from the "1000 genome" reference population were used as reference. The software BEAGLE v3.3.1 was used for 20-MB regions with 10-MB on each side of the SNP of interest. Imputed genotypes with a dosage R^2 <0.3 were excluded from the analysis. The total number of genotyped and imputed SNPs for GWAS was 9,222,825 and that for the pathway-based analysis was 325,934.

Validation of Imputed Genotypes for the HMMR SNPs in LCLs

We isolated DNA from seven LCLs that, based on imputation, possessed the variant genotype (five heterozygous, two homozygous variant) and from six LCLs homozygous wild-type genotype using the DNeasy Blood and Tissue Kit (QIAGEN, Germany). In order to verify the imputed genotypes, Sanger sequencing was undertaken in order to genotype these three SNPs in

HMMR. Primers were designed using the PrimerQuest application available from the IDT website (www.idtdna.com/Primerquest/Home/ Index). PCR was completed using 100 ng of DNA as template with primers flanking both rs299313 and rs29914. Forward and reverse PCR primers primers were 5'-GTAAGAAAGGCTGGAGCAGAA-3 and 5'-GGGAGCTGTACCCAGAAATG-3'. The primers 5'-GAGGTGGCTTACGCCTATAATC-3' and 5'-GAGACAGGATCTCACTCTGT-3' were used to sequence the PCR product containing the rs299313 and rs299314, respectively. Initial primers for PCR amplification of a region flanking rs299293 were 5'-TTCCATTGCCAGCTCCTTAG-3' and 5'-GATCTTCTATCTCACTGGCTGTT -3. Sanger sequencing using the following primer, 5'-ATGTTGGTCATGCTGGTCTC-3' were performed to verify the rs299293 genotype sequence. All samples were treated with the reagent Exo-SAP-IT® (Affymetrix, Santa Clara, CA) prior to sending samples for sequencing.

2 Supplementary Figures

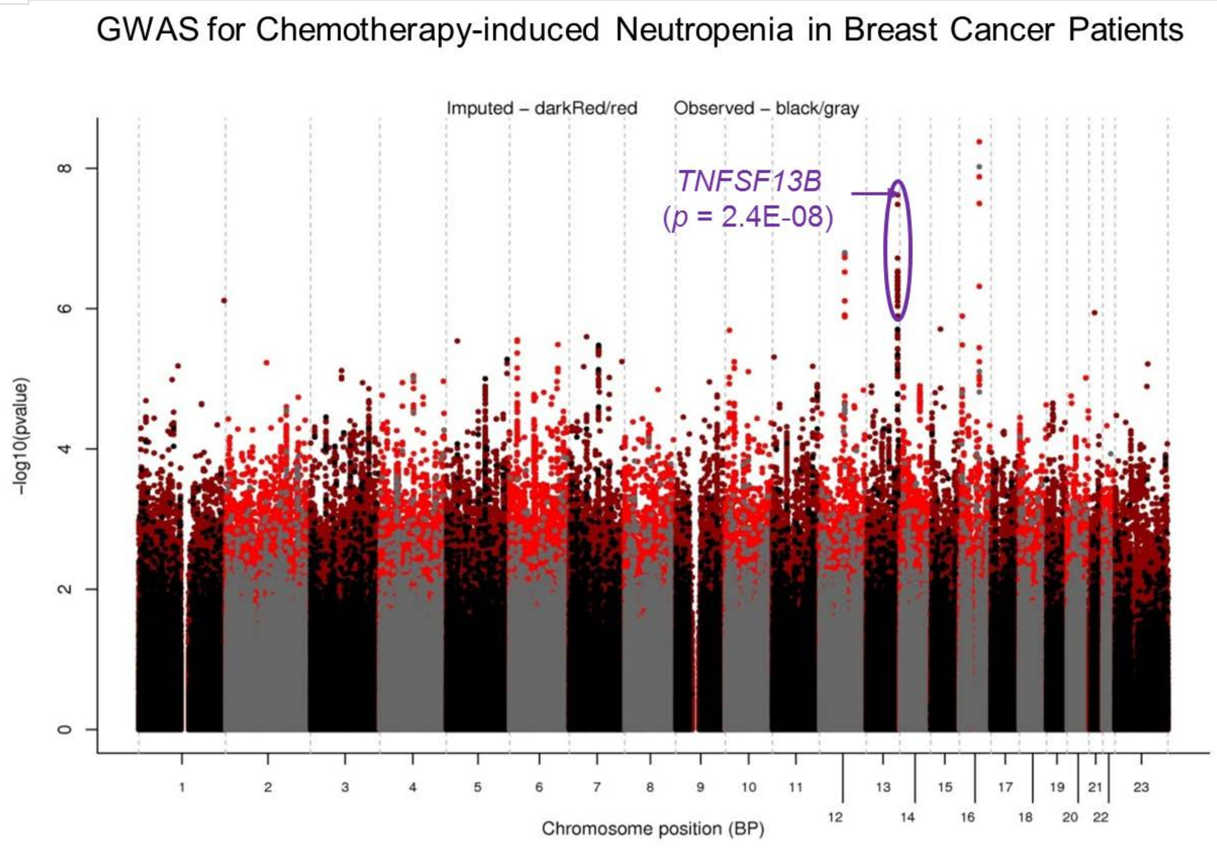

Supplementary Figure S1. Manhattan Plot of GWAS for Chemotherapy-induced Neutropenia in Breast Cancer Patients. A total of 3252 genotyped subjects with covariate information were included in the GWAS analysis. Cases were required to have Grade 3 or 4 toxicity according to

the National Cancer Institute (NCI)'s Common Terminology Criteria for Adverse Events v3.0. SNPs included in the GWAS analysis were genotyped by using the Illumina Human Omni Express Beadchip and were imputed based on the 1K Genomes data. SNP signals, with lowest *p* value of 2.4E-08, which mapped to the *TNFSF13B* on chromosome 13 were identified that were associated with chemotherapy-induced neutropenia.

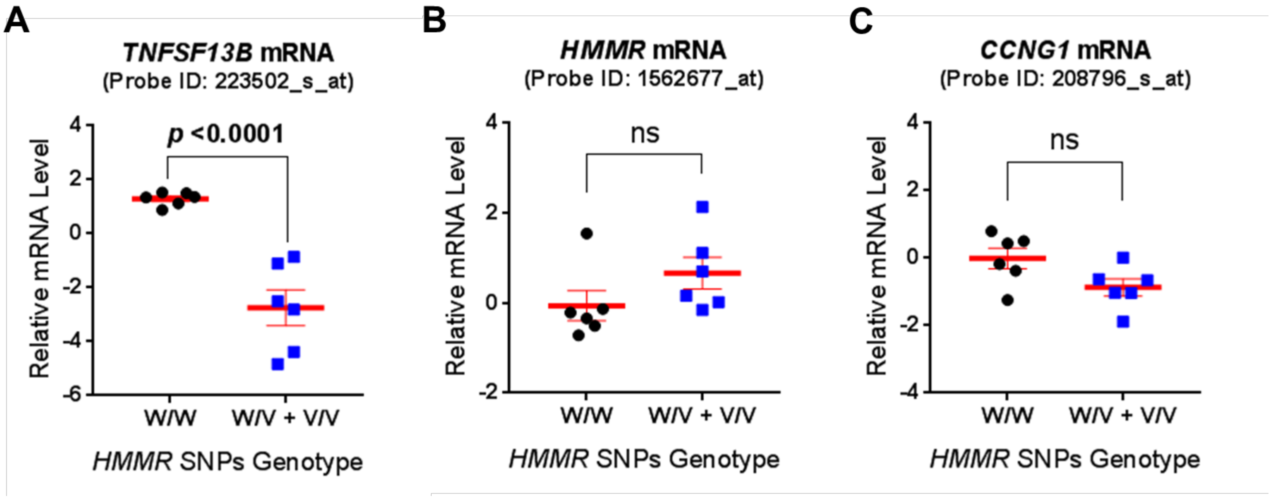

Supplementary Figure S2. The mRNA Levels of (**A**) *TNFSF13B*, (**B**) *HMMR* and (**C**) *CCNG1* in LCLs used in the Cytotoxicity Assay. The mRNA level was determined by the Affymetrix U133 2.0 Plus GeneChip expression array with specific probes as specified. The mRNA levels were grouped based on the genotype of *HMMR* SNPs (rs299293, rs299313, and rs299314), and compared between wild type (W/W) and heterozygous variant (W/V) plus homozygous variant (V/V, n = 2) genotypes by unpaired *t* test. Values are Mean ± S.E.M. A p value < 0.05 was considered statistically significant. ns = not significant.

3 Supplementary Tables

See attached Excel files for Supplementary Table S1 to S6.
